# The Impact of the Coronavirus Disease 19 Pandemic on Early Pregnancy Outcomes Among Patients Undergoing *In Vitro* Fertilization Treatment

**DOI:** 10.1089/whr.2021.0054

**Published:** 2021-10-04

**Authors:** Devora Aharon, Dmitry Gounko, Joseph A. Lee, Alan B. Copperman, Eric Flisser

**Affiliations:** ^1^ Department of Obstetrics, Gynecology, and Reproductive Sciences, Icahn School of Medicine at Mount Sinai, New York, New York, USA.; ^2^ Reproductive Medicine Associates of New York, New York, New York, USA.

**Keywords:** Coronavirus, COVID-19, early pregnancy, pregnancy loss, miscarriage

## Abstract

***Objective:*** To determine if pregnancy rates (PRs) or pregnancy loss rates (PLRs) were altered in patients undergoing single, euploid frozen-thawed embryo transfer (FET) during the initial peak of the Coronavirus Disease 19 (COVID-19) pandemic.

***Materials and Methods:*** This was a retrospective cohort study performed in a single academic center. Patients undergoing single, euploid FET cycles from January to May 2017–2020 were included. Cycles with FET performed in January–May of 2020 (“COVID-surge cohort”) were compared to cycles with FET performed in January–May of 2017–2019 (“pre-COVID cohort”). Pregnancy rate (PR), clinical pregnancy rate (CPR), pregnancy loss rate (PLR), and clinical pregnancy loss rate (CLR) were compared between the cohorts.

***Results:*** A total of 2629 single, euploid FET cycles were included: 2070 from January to May, 2017–2019 and 559 from January to May 2020. PR was similar when comparing FET performed from January to May 2020 (COVID-surge) to those performed from January to May, 2017–2019 (pre-COVID) (77.6% vs. 73.7%, *p* = 0.06), while CPR was higher among the COVID-surge compared to the pre-COVID cohort (65.5% vs. 60.0%, *p* = 0.02). No differences were seen in PLR and CLR among the COVID-surge and pre-COVID cohorts (28.3% vs. 32.0%, *p* = 0.08; 15.0% vs. 16.5%, *p* = 0.50). PR, CPR, PLR, and CLR were similar when comparing individual months between the cohorts. Adjusted analysis showed no differences in PR, CPR, PLR, or CLR when comparing the cohorts overall or when comparing corresponding individual months in the two time periods.

***Conclusion:*** PRs and PLRs were not decreased when SARS-CoV-2 transmission was widespread in our geographic area, suggesting that high COVID-19 transmission does not compromise early pregnancy outcomes.

## Introduction

The global pandemic of SARS-CoV-2 and the Coronavirus Disease 19 (COVID-19) that it causes have reshaped access to health care, including to reproductive health services. Today, there is an increase in the use of telemedicine and incorporation of vigilant practices regarding social distancing, ample use of personal protective equipment (PPE), and consistent decontamination protocols to minimize spread of COVID-19 infection. However, the impact of COVID-19 infection on pregnant women and their fetuses is still largely unknown. Assisted reproductive technology (ART) treatment remains an essential form of medicine, yet patients and providers have expressed concern regarding continuing use of ART and initiating pregnancies during this time.^1–4^

Early reports regarding COVID-19 infection in pregnancy have demonstrated a possible associated increase in preterm delivery, but low likelihood of vertical transmission.^4–10^ The majority of data to date, however, is derived from patients who were in later stages of pregnancy.^7–15^ Whether the SARS-CoV-2 virus or any related social/psychological impact of the pandemic may affect early pregnancy outcomes is currently unknown.

The pandemic's initial peak in the Northeast region of the United States occurred in April 2020. During this period, the prevalence of COVID-19 infection among New York City residents was estimated to be 22.7%, consisting largely of asymptomatic infection.^16^ Given the high rate of infection and concerns about SARS-CoV-2 pathogenesis, an increase in early pregnancy loss might have been expected in the community during this time period if SARS-CoV-2 contributed to adverse early pregnancy outcomes. In the general population, early pregnancy failure is often underreported or undetected, and public health data may therefore be insufficient to inform about the pathogenicity of SARS-CoV-2. Among patients undergoing ART treatment, on the other hand, pregnancy rates (PRs) and pregnancy loss rates (PLRs) are closely tracked and therefore can provide some insight.

The objective of this study was to assess the impact of the COVID-19 pandemic on PRs and PLRs for patients undergoing ART treatment during the initial phase of the pandemic in New York City.

## Materials and Methods

### Study design

This retrospective cohort study was conducted at a single, academic tertiary care center with satellite offices throughout the New York metropolitan area. All single, euploid frozen-thawed embryo transfer (FET) cycles that were performed from January through May of each year of 2017 through 2020 were assessed. Single, euploid FET cycles that occurred during the early months of the COVID-19 pandemic (January–May of 2020, “COVID-surge cohort”) were compared to those that occurred during the corresponding months in the pre-COVID time period (January–May of 2017–2019, “pre-COVID cohort”). This study was approved by an academic Institutional Review Board with a waiver of consent for retrospective analysis of deidentified data.

### *In vitro* fertilization (IVF) protocols

Controlled ovarian stimulation was performed as previously described.^17,18^ Ovarian follicle growth was measured by transvaginal ultrasound (TVUS), and when two or more follicles reached a mean diameter ≥18 mm, recombinant or purified human chorionic gonadotropin, leuprolide acetate, or a combination of the two was used to induce final oocyte maturation. TVUS-guided oocyte retrieval was performed 36 hours later. Intracytoplasmic sperm injection was performed on metaphase II oocytes. Trophectoderm biopsy was performed on days 5, 6, or 7 of embryo development once embryos achieved an adequate morphologic grade (≥4BC modified Gardner morphological score), and then embryos were vitrified as previously described.^19^ Preimplantation genetic testing for aneuploidy was performed using Next Generation Sequencing as described previously.^19^

Endometrial preparation was performed with oral estradiol twice daily for 4 days followed by three times daily.^19^ TVUS was used to assess endometrial thickness and echotexture. When a minimum measurement of endometrial thickness of 7 mm was achieved, daily progesterone was administered *via* intramuscular or oral and vaginal routes. Embryo thawing and transfer were performed after 5 days of progesterone supplementation.

Serum human chorionic gonadotropin (hCG) levels were analyzed 9 days after embryo transfer was performed and repeated 2 days later if positive (serum hCG ≥2.5 mIU/mL). Transvaginal ultrasonography was performed 1 week later and then weekly until discharge from the practice at 8–9 weeks' gestational age. Clinical pregnancy was defined as the presence of a gestational sac on TVUS in the presence of rising hCG.

### COVID-19 protocols

In accordance with guidance set forth by the American Society for Reproductive Medicine (ASRM), at the height of the pandemic, our center stopped initiating *in vitro* fertilization and FET cycles in late March of 2020 and restarted in late May.^1^ Upon resuming services, all patients were screened with temperature checks and symptom and exposure questionnaires. Cycles were cancelled for patients with a positive screen. Modifications were made to laboratory protocols, PPE use, and timing of procedures and patient visits to incorporate vigilant sterilization and distancing techniques.

### Outcomes

The primary aim of our study was to assess the PR, defined as cycles with hCG ≥2.5 mIU/mL, clinical pregnancy rate (CPR), defined as the observed presence of a gestational sac, PLR, which included biochemical or clinical pregnancy losses, and clinical pregnancy loss rate (CLR), clinical pregnancies that were spontaneously aborted, from single, euploid FETs performed during the initial peak of COVID-19 infections compared to those performed during the corresponding pre-COVID time period. Secondary aims were to analyze the same outcomes by individual month in the same timeframe. Demographic and cycle characteristics were compared, including age, oocyte age, body mass index (BMI), serum Anti-Müllerian Hormone (AMH) level, and endometrial thickness.

### Statistical analysis

Differences among categorical variables and continuous variables were examined using Chi-Squared tests and Student's *t*-tests, respectively. Gaussian distribution assumptions were assessed using the Shapiro–Wilk test, and differences among nonparametric variables were established using the Wilcoxon Rank-Sum test. Multivariable logistic regression was performed to predict the probability of PRs and PLRs and control for confounding variables. Potential confounders assessed included patient age, oocyte age, serum AMH, BMI, and endometrial thickness. A *p*-value of 0.05 was considered statistically significant. SAS Studio 3.8 was used for statistical analysis.

## Results

A total of 2629 single, euploid FET cycles were included in the study: 559 from January through May of 2020 (COVID-surge) and 2070 from January through May of 2017–2019 (pre-COVID). Baseline characteristics, including age, oocyte age, AMH, BMI, and endometrial thickness, were similar between the groups (Table 1). No significant differences were found in single, euploid FET PRs between the COVID-surge and pre-COVID cohorts (77.6% vs. 73.7%, OR = 1.24, 95% CI 0.99–1.55, *p* = 0.06). CPR was significantly higher among the COVID-surge compared to the pre-COVID cohort (65.5% vs. 60.0%, OR = 1.26, 95% CI 1.04–1.54, *p* = 0.02).

**Table 1.** Demographic Characteristics and Early Pregnancy Outcomes Among Single, Euploid Frozen-Thawed Embryo Transfer Performed in January through May, 2020 Compared to January Through May, 2017–2019

	January–May 2020 (N = 559)	January–May 2017–2019 (N = 2070)	*p*
Age (year)	36.8 ± 4.4	36.9 ± 4.5	0.60
Oocyte age (year)	34.9 ± 4.5	35.1 ± 4.6	0.35
Anti-Müllerian Hormone (ng/mL)	3.15 ± 2.80	3.60 ± 4.04	0.23
Body mass index (kg/m^2^)	24.4 ± 4.7	24.2 ± 4.5	0.34
Endometrial thickness (mm)	9.61 ± 2.24	9.63 ± 2.28	0.77
Pregnancy rate	77.6% (434/559)	73.7% (1526/2070)	0.06
Clinical pregnancy rate	65.5% (366/559)	60.0% (1242/2070)	0.02
Pregnancy loss rate	28.3% (123/434)	32.0% (489/1526)	0.14
Clinical pregnancy loss rate	15.0% (55/366)	16.5% (205/1242)	0.50

No significant differences in PLR (28.3% vs. 32.0%, OR = 0.84, 95% CI 0.66–1.06, *p* = 0.14) or CLRs (15.0% vs. 16.5%, OR = 0.89, 95% CI 0.65–1.24, *p* = 0.50) were observed when comparing single, euploid FET performed in the COVID-surge cohort to those performed in the pre-COVID cohort (Table 1). In addition, no differences were seen in PR, CPR, PLR, or CLR when comparing individual months in the COVID-surge and pre-COVID time periods (Table 2).

**Table 2.** Early Pregnancy Outcomes by Month of Embryo Transfer in 2020 Compared to 2017–2019

Outcome	Month	2020	2017–2019	*p*
Pregnancy rate	January	76.0% (117/154)	72.0% (260/361)	0.35
	February	80.1% (113/141)	73.6% (292/397)	0.12
	March	72.0% (103/143)	71.9% (315/438)	0.98
	April	75% (3/4)	73.9% (294/398)	0.96
	May	83.8% (98/117)	76.7% (365/476)	0.10
Clinical pregnancy rate	January	64.9% (100/154)	59.6% (215/361)	0.25
	February	66.0% (93/141)	61.0% (242/397)	0.29
	March	62.2% (89/143)	59.1% (259/438)	0.51
	April	75% (3/4)	60.6% (241/398)	0.56
	May	69.2% (81/117)	59.9% (285/476)	0.06
Pregnancy loss rate	January	30.8% (36/117)	31.2% (81/260)	0.94
	February	30.1% (34/113)	27.4% (80/292)	0.59
	March	23.3% (24/103)	31.1% (98/315)	0.13
	April	0% (0/3)	31.6% (93/294)	0.24
	May	29.6% (29/98)	37.5% (137/365)	0.15
Clinical pregnancy loss rate	January	19% (19/100)	16.7% (36/215)	0.62
	February	15.1% (14/93)	12.4% (30/242)	0.52
	March	11.2% (10/89)	16.2% (42/259)	0.26
	April	0% (0/3)	16.6% (40/241)	0.44
	May	14.8% (12/81)	20.0% (57/285)	0.29

Multivariable logistic regression showed no differences in odds of pregnancy or clinical pregnancy in the COVID-surge cohort compared to the pre-COVID cohort when controlling for oocyte age, AMH, BMI, and endometrial thickness (PR: OR = 1.26, 95% CI 0.98–1.62, *p* = 0.07; CPR: 1.24, 95% CI 0.99–1.54, *p* = 0.06). No differences were seen in odds of pregnancy loss or clinical pregnancy loss on adjusted analysis (PLR: OR = 0.79, 95% CI 0.61–1.03, *p* = 0.08; CLR: OR 0.75, 95% CI 0.51–1.09, *p* = 0.13). Odds of pregnancy, clinical pregnancy, pregnancy loss, and clinical pregnancy loss were similar when comparing each corresponding month during the two time periods, controlling for the covariates above (Supplementary Table S1).

## Discussion

The COVID-19 pandemic has placed an unprecedented burden on patients, physicians, and, indeed, the entire health care system. Urgent treatments, including reproductive care, were frequently delayed as scarce resources were temporarily redirected. Restoration of postponed medical care required modifications to workflow, staffing, decontamination protocols, and utilization of PPE. While the patient experience has changed, our study demonstrates that PRs and loss rates were not compromised at the height of the pandemic when SARS-CoV-2 transmission was widespread in our community.

Our study analyzed single, euploid FETs taking place from January through May of 2020. Patients who became pregnant during this period underwent their first trimester of pregnancy during the initial peak of the pandemic in March through May of 2020, a time of high asymptomatic community transmission, and therefore were at risk of unknown exposure to the virus and effects of the pandemic during early gestation. If asymptomatic infection or social/emotional impacts of the pandemic itself impacted early pregnancy, a difference in implantation and early pregnancy loss would be expected to be seen during this time period. By comparing ART treatment outcomes of these patients to those who underwent single, euploid FETs during the same months of 2017–2019, we were able to control for potential seasonal variations in implantation and early PLRs.^20,21^

Data are extremely limited regarding the impact of SARS-CoV-2 on pregnancy loss. While one study found an increased rate of stillbirth ≥24 weeks gestational age during the COVID-19 pandemic compared to the period prior, two other studies failed to replicate this finding.^22–24^ A secondary analysis of a multinational cohort study reported six miscarriages in 388 pregnant patients diagnosed with COVID-19 (2.3%), while a retrospective study found one miscarriage out of 54 pregnancies with confirmed or suspected COVID-19 infection (1.9%).^6,25^ Those studies and others that have evaluated the impact of COVID-19 on pregnancy are comprised mostly of women who were already in the second or third trimester during the early days of high SARS-CoV-2 transmission, while few studies have focused on the impact of COVID-19 on early pregnancy outcomes.^7,11,12,15,26^ One case–control study found no difference in the incidence of COVID-19 infection in women with and without first-trimester spontaneous pregnancy loss, while a retrospective study found no differences in viable and arrested pregnancies among women who presented for a first-trimester viability scan in the COVID and pre-COVID time periods.^27,28^ The inclusion of only detected pregnancies and reported losses may bias the results of these studies. A retrospective study of pregnancies resulting from IVF or intrauterine insemination reported a miscarriage rate of 14.4% from December 2019 to March 2020, although the lack of a comparison group in this study limits the interpretation of this finding.^29^

Our results are consistent with prior data that have not found an increased risk of pregnancy loss in areas of high SARS-CoV-2 prevalence. This is in contrast to data on SARS-CoV-1 (SARS) and Middle Eastern Respiratory Syndrome which, while limited to case series, indicated an elevated risk of pregnancy loss and fetal demise in infected pregnant women of up to 27%–57%.^30–32^ In addition, concerns have emerged regarding the COVID-19 vaccine and early pregnancy outcomes, given the lack of inclusion of pregnant women in vaccine trials.^33^ Until prospective data regarding the COVID-19 vaccine in pregnant women are available, our data may provide a measure of reassurance that the immune response to SARS-CoV-2 does not appear to impact early pregnancy outcomes.

This study is one of the first to focus on early pregnancy outcomes during the primary surge of the COVID-19 pandemic in the New York metropolitan area. Strengths of this study include the large cohort and the use of a control group matched by month. During the study period, screening for SARS-CoV-2 was neither widely available nor clinically recommended, yet asymptomatic infection was widespread.^34^ Only studying patients with positive COVID-19 test results would likely have missed a large number of asymptomatic carriers given the extremely limited availability of screening at the time and would have underestimated the impact of the virus on early pregnancy outcome if any infection, including otherwise asymptomatic infection, contributes to adverse outcomes. Similarly, only studying symptomatic patients could overemphasize the impact of virus infection on adverse pregnancy outcomes. Our study evaluated the entire cohort of potentially exposed patients to assess for a possible occult impact of the virus on early pregnancy. Studying ART patients, in which outcomes are closely tracked, allowed us to capture true rates of early pregnancy failure in contrast to the general population, in which early pregnancy loss may be selectively and under-reported. Restricting our analysis to single, euploid FET removed a number of potential confounders that may impact PRs and PLRs in attempt to isolate the impact of the pandemic on implantation and early clinical pregnancy.

This study has significant limitations. Universal screening for SARS-CoV-2 in our community was not performed due to lack of availability and clinical recommendations against doing so.^34^ Without universal testing, these data cannot definitively exclude a possible impact of SARS-CoV-2 infection on pregnancy loss. Although our center resumed treatments in late May, we continued to delay treatment for patients with known risk factors for severe COVID-19 infection, such as obesity, hypertension, and type II diabetes, which are also known to increase risks for early pregnancy failure. This may account for a higher clinical PR observed in January–May 2020 compared to previous years, which was not significant when controlling for covariates. In addition, embryo transfers were cancelled for patients with a positive screen due to elevated temperature, symptoms, or exposure history. However, these protocols only excluded patients from having embryo transfer; patients may have had exposure or symptoms subsequent to transfer during the first trimester of pregnancy. Given that new cycles were not initiated during the peak of virus transmission and screening protocols were in place upon reopening, our results may suggest that, at the very least, screening patients with temperature checks and symptom/exposure questionnaires may be effective in maintaining established early pregnancy success rates.

## Conclusions

Early pregnancy outcomes were not different during a time of high SARS-CoV-2 infection in our community. These data indicate that even with high rates of asymptomatic transmission, ART may continue to be provided during this pandemic without a detrimental impact on pregnancy outcome. This information may help guide clinics addressing regional surges in virus transmission as they determine whether to continue providing treatment and develop safety protocols. Our findings may also provide reassurance that the currently prevalent, highly infectious Delta variant is unlikely to have an effect on early pregnancy in cases of asymptomatic infection. While additional studies assessing the rate of pregnancy loss in women with confirmed SARS-CoV-2 infection are needed, these data are reassuring that high rates of community COVID-19 transmission do not appear to impact early pregnancy outcomes.

## Supplementary Material

Supplemental data

## Abbreviations Used

AMH: anti-Müllerian hormone
ART: assisted reproductive technology
BMI: body mass index
CLR: clinical pregnancy loss rate
COVID-19: coronavirus disease 19
CPR: clinical pregnancy rate
FET: frozen-thawed embryo transfer
hCG: human chorionic gonadotropin
IVF: *in vitro* fertilization
PLR: pregnancy loss rate
PPE: personal protective equipment
PR: pregnancy rate
TVUS: transvaginal ultrasound
