# Supplementary material for: The Impact of the Coronavirus Disease 19 Pandemic on Early Pregnancy Outcomes Among Patients Undergoing In Vitro Fertilization Treatment
Source: Womens Health Rep (New Rochelle). 2021 Oct 4;2(1):473–8. doi: 10.1089/whr.2021.0054 (PMC8617588; doi:10.1089/whr.2021.0054)
Supplement: Supplemental data [file Supp_TableS1.docx]

Supplementary Table 1. Adjusted odds of pregnancy, clinical pregnancy, pregnancy loss, and clinical pregnancy loss by month in 2020 vs. 2017-2019, controlling for oocyte age, AMH, BMI, and endometrial thickness

| Outcome | Month | Adjusted OR | 95% CI | P value |
| --- | --- | --- | --- | --- |
| Pregnancy | January | 1.41 | 0.85-2.33 | .19 |
|  | February | 1.52 | 0.90-2.57 | .12 |
|  | March | 1.01 | 0.63-1.64 | .95 |
|  | April | 1.08 | 0.11-10.8 | .95 |
|  | May | 1.39 | 0.78-2.48 | .27 |
| Clinical pregnancy | January | 1.41 | 0.90-2.20 | .14 |
|  | February | 1.29 | 0.82-2.02 | .27 |
|  | March | 1.15 | 0.74-1.78 | .54 |
|  | April | 2.41 | 0.24-23.9 | .45 |
|  | May | 1.17 | 0.73-1.87 | .52 |
| Pregnancy loss | January | 0.82 | 0.47-1.41 | .46 |
|  | February | 1.08 | 0.97-1.10 | .77 |
|  | March | 0.71 | 0.40-1.25 | .23 |
|  | April | <0.01 | <0.01- >999.99 | .99 |
|  | May | 0.73 | 0.43-1.26 | .26 |
| Clinical pregnancy loss | January | 0.96 | 0.45-2.03 | .90 |
|  | February | 1.18 | 0.52-2.65 | .69 |
|  | March | 0.70 | 0.31-1.59 | .40 |
|  | April | <0.01 | <0.01- >999.99 | .98 |
|  | May | 0.49 | 0.21-1.14 | .10 |
